# Piloting a surveillance system for HIV drug resistance in the European Union

**DOI:** 10.2807/1560-7917.ES.2019.24.19.1800390

**Published:** 2019-05-09

**Authors:** Marita JW van de Laar, Arnold Bosman, Anastasia Pharris, Emmi Andersson, Lambert Assoumou, Eva Ay, Norbert Bannert, Barbara Bartmeyer, Melissa Brady, Marie-Laure Chaix, Diane Descamps, Kenny Dauwe, Jannik Fonager, Andrea Hauser, Maja Lunar, Maria Mezei, Martha Neary, Mario Poljak, Ard van Sighem, Chris Verhofstede, Andrew J Amato-Gauci, Eeva K Broberg

**Affiliations:** 1 MJW van de Laar Consultancy, Zennewijnen, The Netherlands; 2 Transmissible, Houten, The Netherlands; 3 European Centre for Disease Prevention and Control (ECDC), Stockholm, Sweden; 4 Division of Clinical Microbiology, Department of Laboratory Medicine, Karolinska Institute, Stockholm, Sweden; 5 Department of Clinical Microbiology, Karolinska University Laboratory, Stockholm, Sweden; 6 INSERM, Sorbonne Université, Institut Pierre Louis d’Epidémiologie et de Santé Publique, Paris, France; 7 National Public Health Institute, Department of Retroviruses, Budapest, Hungary; 8 Robert Koch Institute, Berlin, Germany; 9 Health Service Executive (HSE), Health Protection Surveillance Centre, Dublin, Ireland; 10 Laboratoire de Virologie, APHP, Saint Louis Hospital, INSERM UMR944, Paris, France; 11 Paris Diderot University, Paris, France; 12 Laboratoire de Virologie, Bichat-Claude Bernard University Hospital, INSERM UMR_1137, Paris, France; 13 Aids Reference Laboratory, Department of Diagnostic Sciences, Ghent University, Ghent, Belgium; 14 Virus and Microbiological Special Diagnostics, Infectious Disease Preparedness, Statens Serum Institut, Copenhagen, Denmark; 15 Institute of Microbiology and Immunology, Faculty of Medicine, University of Ljubljana, Ljubljana, Slovenia; 16 University College Dublin (UCD), National Virus Reference Laboratory, Dublin, Ireland; 17 Stichting HIV Monitoring, Amsterdam, The Netherlands

**Keywords:** HIV/AIDS, resistance, surveillance, pilot study

## Abstract

**Background:**

A steady increase in HIV drug resistance (HIVDR) has been demonstrated globally in individuals initiating first-line antiretroviral therapy (ART). To support effective use of ART and prevent spread of HIVDR, monitoring is essential.

**Aim:**

We piloted a surveillance system for transmitted HIVDR to assess the feasibility of implementation at the European level.

**Method:**

All 31 countries in the European Union and European Economic Area were invited to retrospectively submit data on individuals newly diagnosed with HIV in 2015 who were tested for antiviral susceptibility before ART, either as case-based or as aggregate data. We used the Stanford HIV database algorithm to translate genetic sequences into levels of drug resistance.

**Results:**

Nine countries participated, with six reporting case-based data on 1,680 individuals and four reporting aggregated data on 1,402 cases. Sequence data were available for 1,417 cases: 14.5% of individuals (n = 244) showed resistance to at least one antiretroviral drug. In case-based surveillance, the highest levels of transmitted HIVDR were observed for non-nucleoside reverse-transcriptase inhibitors (NNRTIs) with resistance detected in 8.6% (n = 145), followed by resistance to nucleoside reverse-transcriptase inhibitors (NRTI) (5.1%; n = 85) and protease inhibitors (2.0%; n = 34).

**Conclusion:**

We conclude that standard reporting of HIVDR data was feasible in the participating countries. Legal barriers for data sharing, consensus on definitions and standardisation of interpretation algorithms should be clarified in the process of enhancing European-wide HIV surveillance with drug resistance information.

## Background

The global scale-up of antiretroviral treatment (ART) has led to considerable reductions in HIV-related morbidity and mortality. Increasing the proportion of patients who achieve viral suppression during treatment will further reduce HIV transmission rates [1]. However, HIV drug resistance (HIVDR) poses a threat to the long-term success of ART and to the elimination of HIV/AIDS [2]. To address this issue, the World Health Organization (WHO) published a Global Action Plan on HIV Drug Resistance [3]. One of the five strategic objectives focuses on monitoring and surveillance to ensure access to the most effective drugs. Preventing and managing the occurrence of HIVDR is a key component of a comprehensive and effective HIV response and should be integrated into broader efforts to ensure sustainability. It is essential that actions to monitor, prevent and respond to HIVDR are implemented at clinical, programme and policy level, targeting the various drivers of HIVDR.

At present, there is no European-level surveillance of HIVDR, although individual clinicians request sequence-based data in patients newly diagnosed with HIV before antiretroviral treatment initiation (or in case of treatment failure) to guide the choice of the first-line ART as recommended by the European AIDS Clinical Society [4]. Because of its impact on public health and clinical treatment guidelines, transmitted drug resistance (TDR) is the primary focus of HIVDR surveillance at the European level, e.g. in the SPREAD project [5]. The prevalence of TDR is an important indicator to inform national and European Union (EU) guidance on therapy initiation for newly diagnosed HIV patients. Systematic HIVDR surveillance is the best tool to deliver timely and representative information on a regular basis. Such surveillance will also provide essential information on the most adequate pre- and post-exposure prophylaxis regimens and treatment in general.

A recent survey within the European HIV surveillance network revealed that some countries publish their national HIVDR data in surveillance reports or in scientific publications [6]. However, it also indicated that countries use different methods to define and select study populations and this may complicate the interpretation and comparison of national HIVDR prevalence data. International comparison of HIVDR results may also be hampered by different methods for the interpretation of drug resistance mutations. Although a number of EU countries have the technical and epidemiological capacity to monitor HIVDR, there is currently no common surveillance cycle, nor a common framework to interpret findings from HIVDR monitoring. Here, we describe a pilot study aimed at enhancing HIV surveillance with information on TDR and assessing its feasibility for implementation at the European level.

## Methods

In September 2017, the European Centre for Disease Prevention and Control (ECDC) invited nationally appointed HIV experts from all 31 countries in the EU and European Economic Area (EEA) to participate in the pilot study. Countries were invited to participate if they had submitted epidemiological HIV surveillance data (year of diagnosis 2015) to The European Surveillance System (TESSy). We asked the countries to submit retrospectively HIVDR data that were already available at the national level and to contribute to the development of a reporting protocol for the pilot surveillance system. The reporting protocol specified three reporting options: (i) case-based data with HIV sequences, (ii) case-based data with mutation codes or drug resistance interpretations, or (iii) aggregate data. Mutations codes were selected by the countries based on their own sequencing results. Reported additional variables included demographic, epidemiological and clinical data. Aggregate data were requested by sex (male/female), transmission route and main drug class.

We defined the population of interest for the HIVDR surveillance pilot as ‘newly diagnosed treatment-naïve HIV patients tested before initiating HIV treatment for susceptibility to any of the 22 available antiretroviral (ARV) drugs in the four main drug classes’. In this study, we only collected information on diagnoses with HIV-1. In the context of this pilot study, pre-exposure prophylaxis (PrEP) was not considered treatment, but cases who at some point in time received PrEP were included. HIVDR was defined as any mutation or combination of mutations that, according to the Stanford HIV drug resistance database (HIVdb) [7], results in low, intermediate or high-level resistance to currently available drug classes: non-nucleoside reverse transcriptase inhibitor (NNRTI), nucleoside reverse transcriptase inhibitor (NRTI), protease inhibitor (PI) and integrase inhibitor (INI).

In this pilot, the Stanford HIVdb version 8.4 algorithm was used, which defines the interpretation of the submitted sequences [7]. HIVdb is an expert web-interface system that accepts user-submitted HIV-1 sequences and returns inferred levels of resistance to 22 ARV drugs. HIVdb is regularly updated and new resistance patterns are included. The HIVdb system assigns a drug penalty score to each drug resistance mutation (DRM; consisting of an amino acid position number and the amino acid change from the wildtype to the mutation, e.g. V106I in the reverse transcriptase protein); for each sequence, the total score for a drug is derived by adding the scores of each DRM associated with resistance to that drug. The programme reports one of the following five levels of inferred drug resistance (defined as ‘drug resistance interpretation’): susceptible, potential low-level resistance, low-level resistance, intermediate resistance and high-level resistance. In this pilot, we considered ‘potential low-level resistance’ as ‘susceptible’.

For specific selection of TDR, the Stanford database also presents a list of surveillance drug resistance mutations (SDRM list; mutation codes are available from [8]) that contains 93 mutations, including 34 NRTI-resistance mutations at 15 reverse transcriptase (RT) gene positions, 19 NNRTI-resistance mutations at 10 RT positions and 40 PI-resistance mutations at 18 protease gene positions [9,10]. This list does not include integrase resistance mutations.

Country representatives submitted data online, as comma-separated value files, to a secure data exchange platform (Voozanoo, EpiConcept) which ensured data security compliant with European standards for processing health data. We checked the uploaded files manually for compliance with the correct format and merged the data into a single spreadsheet. We parsed text files with sequence data, linked to unique record identifiers, through the online Stanford HIVdb algorithm and merged the output into the datasheet to allow comparison of drug resistance interpretation with the original submissions.

After data submission, we interviewed the national experts to assess the feasibility of reporting and to evaluate the pilot HIVDR surveillance system. The interviews aimed to understand the limitations, barriers and challenges that were encountered during data preparation and submission. Also, to assess the comparability of the data from different European countries, an estimation of the national coverage of the HIVDR data was requested from the individual countries. Participants received the questionnaire in advance to prepare for the interviews. Individual summary reports with the information from the questionnaire and the interviews were validated by all countries.

## Results

### Data submission and overall prevalence of HIV drug resistance

Nine of 31 countries participated in the pilot study, six reported case-based data and four aggregated data (one country reported both). Table 1 shows the data submission by country: 1,680 case-based records, 97 aggregate data records, and two sets with aggregate data from France. The data submitted for this pilot represent a proportion of the total number of new HIV diagnoses in 2015 in the participating countries, ranging from 5% in Hungary to 60% in Sweden and Ireland (Table 1). The completeness of reporting ranged from 21% for integrase sequences to 100% for sex and prior ARV treatment.

**Table 1:** Number of records submitted per country, HIV drug resistance pilot surveillance, European Union, 2017 (n = 9 countries)

Country	Reported records for HIVDR pilot surveillance (year 2015)			New HIV diagnoses in 2015 [15]	
	Case-based records	Aggregate records	Number of HIV diagnoses	n	% included in pilot
Belgium	472			1,001	47
Germany	618			3,674	17
Hungary	14			271	5
The Netherlands	277			802	35
Slovenia	15			48	31
Sweden	284	18	284	447	64
Denmark		64	118	277	43
France		0^a^	707	3,943	18
Ireland		15	293	486	60
**Total**	**1,680**	**97**	**1,402**	**10,949**	**15 **

HIV: human immunodeficiency virus; HIVDR: HIV drug resistance;

^a^ Data were excluded from analysis as submission was not according to the reporting protocol.

The six countries that provided case-based surveillance data also reported sequences: 1,417 records included one or more protease or reverse transcriptase sequences. Five countries submitted sequences for all individual records; Sweden provided sequences for TDR cases only. Table 2 presents the number of submitted sequences, the mutation codes (from Stanford HIVdb) and the overall observed TDR prevalence by country. France submitted only two sets with outcome tables presenting aggregated cases by sex and was therefore excluded from the analysis.

**Table 2:** Submitted sequences, mutation codes and proportion with transmitted drug resistance, case-based reporting, HIV drug resistance surveillance pilot, European Union, 2017 (n = 6 countries)

Country	Records submitted	Sequences submitted	Any mutation code	% TDR	95% CI
Belgium	472	472	74	15.7	12.5–19.3
Germany	618	618	114	18.4	15.5–21.7
Hungary	14	14	1	7.1	0.2–33.9
The Netherlands	277	277	34	12.3	8.7–16.7
Slovenia	15	15	0	0.0	0–21.8
Sweden^a^	284	21	21	7.4	4.6–11.1
**Total**	**1,680**	**1,417**	**244**	**14.5**	**12.9–16.3**

CI: confidence interval; HIV: human immunodeficiency virus; TDR: transmitted drug resistance.

^a^ Sweden only submitted sequences for TDR cases.

Table 3 presents the overview of reported TDR in case-based surveillance (six countries) and aggregate surveillance (three countries). TDR in the case-based surveillance was calculated from reported resistance mutation codes and resistance interpretations, combining low, intermediate and high resistance for each of the four drug classes. TDR in the aggregate surveillance is as reported by the countries. The overall TDR was 14.5% among case-based surveillance and was higher than the 11.9% overall TDR reported in aggregate format.

**Table 3:** Number of cases (and proportion of tested) with mutations in newly diagnosed HIV cases, HIV drug resistance pilot surveillance, European Union, 2017 (n = 9 countries)

Drug class	Case-based surveillance (6 countries)		Aggregate surveillance (3 countries)		Total (combined for 9 countries)	
	n	%	n	%	n	%
Total number tested	1,674	100	1,118	100	2,792	100
HIVDR any class^a^	244	14.6	133	11.9	377	13.5
NRTI	85	5.1	58	5.2	143	5.1
NNRTI	145	8.7	71	6.4	216	7.7
PI	34	2.0	31	2.8	64	2.3
INI^b^	10		2		12	

DRM: drug resistance mutation; HIV: human immunodeficiency virus; HIVDR: HIV drug resistance; INI: integrase inhibitor; NRTI: nucleoside reverse transcriptase inhibitor; NNRTI: nucleoside reverse transcriptase inhibitor; PI: protease inhibitor.

^a^ At least one DRM reported as low, intermediate or high in any of the groups (NRTI, NNRTI, PI or INI).

^b^ It is unknown how many individuals were tested for INI resistance, so no percentage is calculated.

This table displays number of cases; because six duplicates were reported, the number of cases is different from the number of records reported in Table 2, column ‘Records submitted’.

### Congruence – methodological variations

Comparison of the reported resistance interpretation with the results of our sequence analysis showed an overall 98.8% congruence with a wide range of 36–100% (data not shown). The lowest observed congruence was among 11 cases with intermediate NNRTI resistance according to sequence analysis (36%).

Comparison of the reported mutation codes with the results of our sequence analysis showed high levels of congruence. For example, between 95% and 96% of the reported NRTI, NNRTI and PI mutation codes were identical to the mutation codes that we generated with the sequence analysis of the Stanford HIVdb algorithm (data not shown). However, in 32 (16%) of the 200 records with reported mutation codes, the Stanford algorithm identified additional mutation codes.

### Expert interviews

The national data source for HIVDR test results was the same as for the epidemiological HIV surveillance in Germany, Hungary, and the Netherlands. For the other six countries the data source was different because the HIVDR specimens and test results were mostly obtained through a collaboration between clinics or laboratories.

The sampling frame for HIVDR testing was comprehensive for Belgium, France, Germany, Hungary, Ireland and Sweden, it was sentinel for Denmark and ‘other’ for the Netherlands and Slovenia. In the interviews, estimates for the national coverage of HIVDR sampling and testing ranged from 5% to 80% of the total population of newly diagnosed HIV patients. All countries except France and Hungary stated that the test results were representative for the true TDR prevalence in their country. Countries considered the population captured in the HIV surveillance system representative for the national population of newly diagnosed HIV patients. France and Hungary did not describe all newly diagnosed patients that were tested for drug resistance and coverage was therefore too low to produce reliable TDR prevalence rates. All other countries argued that the sampling framework or the selected reference laboratories did not introduce bias towards certain sub-populations or drug classes. However, it was noted that data for Ireland may include some cases of acquired drug resistance in patients who were newly diagnosed in Ireland but who may have been previously treated abroad.

The countries followed different internal procedures to organise and carry out the HIVDR testing: Denmark, Germany, Hungary, Ireland, Slovenia and Sweden reported that HIVDR testing was done at the central level, including the analyses and interpretation. In Belgium, France and the Netherlands, the HIVDR test results were collected and analysed at the central level, but the actual sequences were obtained from regional centres or laboratories.

The results of HIVDR testing could be linked to an individual HIV diagnosis in the epidemiological surveillance in Belgium, Denmark, France, Hungary, the Netherlands and Slovenia. In most cases, this was done by a unique identifier requiring validation by the public health institute. In three countries, the linkage was either explicitly forbidden by data protection and patient safety regulations (Sweden) or was under review (Germany and Ireland).

## Discussion 

This HIVDR surveillance pilot was a first study on the feasibility of setting up routine HIVDR monitoring in the EU/EEA. Results from the pilot suggest differences among the nine participating countries with respect to methodology (e.g. selection of population under surveillance, type of resistance studied) and application of standards (e.g. use of mutation code, interpretation of the SDRM list vs sequence analysis using the Stanford HIVdb). As it is likely that signals of emerging or increasing HIVDR could start in a single country or in a specific sub-population, it is essential that all countries and sub-populations should be represented in future HIVDR surveillance. This was already the case for most countries participating in specific, EU-wide studies such as SPREAD [5]. Comparison of continuous data is key to detection of unusual signals and lack of harmonisation is a barrier for accurate comparison of data. We consider this a valid argument for EU-wide coordination and harmonisation of HIVDR monitoring.

A key question for future EU-wide HIVDR surveillance is whether data should be collected in case-based or aggregate format. Case-based surveillance that includes sequences would allow the greatest flexibility: it allows adjustment to future changes in resistance patterns and (re-)analysis of the data. Newly discovered resistance mutations can be added to the analytical algorithm and applied to historical data, to allow a more reliable description of trends over time, with less risk of surveillance artefacts from re-classification of resistance mutations. This pilot study showed high levels of congruence: almost all sequence-detected mutations were also reported through the national interpretations. However, the sequences also provided additional relevant mutations. Standardised drug resistance interpretation among countries is crucial and surveillance using sequences will be more accurate as it avoids coding errors that might occur in a system that collects mutation codes or interpretations derived from mutation codes.

From a conceptual point of view, we see in the above sufficient arguments in favour of an EU-coordinated HIVDR surveillance system that collects case-based data with sequences. However, there are considerable challenges for implementation of such a molecular surveillance system at EU level. Technical issues identified in this pilot will need to be addressed. The semi-automatic approach in this pilot identified a small number of sequences that could not be parsed and therefore a uniform quality assessment of sequence data would need to be applied before sequence submission.

Even though we believe that the technical solutions will not be trivial, such barriers may be overcome relatively easily. Patient confidentiality, data protection, data ownership and sharing issues on the other hand, were mentioned by all countries as major obstacles for submission of case-based data with sequences. Although the pilot demonstrated that countries were technically able to submit case-based data and sequences, not all had permission to do so as legal obstacles prevented the linking of molecular data and data submission. Among the countries that did submit sequences, most indicated that this could only be done for the pilot and that sequence data had to be destroyed at closure of the project. This was even mentioned for aggregated data sets. Furthermore, access to the sequence data by third parties must be controlled. The intended use of sequence data should be clearly defined and sharing of sequence data should be restricted and based on signed agreements and surveillance protocols. Collection of aggregate data sets may reduce patient confidentiality and data protection issues, but it would be more difficult to analyse the data across countries. The pilot also showed that preparing aggregate data sets is error-prone. An alternative would be to collect only the mutation codes or resistance interpretations, provided that all countries would agree on common resistance interpretation rules and that countries agree to submit case-based data.

The overall TDR prevalence in this pilot (13.5%) was higher than previously reported for European countries [5]. This could be due to the use of different case definition, different methods for calculating and interpreting the resistance mutations or the use of different cut-off values for resistance, or it could reflect the true TDR prevalence in the participating countries. Feedback from participating countries on the observed higher TDR prevalence suggests that the difference was probably caused by using different definitions of resistance and including integrase resistance. Previous reports from most countries were based on the SDRM list, while we used the Stanford HIVdb algorithm for the analysis. The latter is regularly updated and includes polymorphisms which are not included in the SDRM list. Moreover, this pilot study was meant to test feasibility of reporting, and results from the limited data collection may not be interpreted as the true EU prevalence. Representativeness of the surveillance data was assessed only in qualitative interviews with country representatives. If European HIVDR surveillance is considered in the future, then it is recommendable to perform a systematic assessment of representativeness of the data sources.

Belgium reported 9.9% overall TDR in 2015 [11] which is lower than the 15.7% in this pilot. This is most probably due to using the WHO SDRM list from 2009 [10] rather than the Stanford HIVdb list. The observed TDR prevalence in Germany and Sweden was higher than in previous reports [12,13]. However, recent data from Sweden showed results comparable to our findings [14]. In the German data, discrepancy was found between reported resistance interpretation and calculated resistance level in 25 of the 114 cases, nine of which had integrase resistance only. However, even when discarding these 25 observations, the overall TDR remained higher than expected (14.4%). This too may be explained by the fact that Germany used the 2009 SDRM list in previous HIVDR reports rather than Stanford HIVdb, which has the list of updated SDRM in use and therefore detects higher number of SDRMs than the WHO SDRM list from 2009.

## Conclusion

From a technical feasibility point of view, this surveillance pilot study can be considered as a success. All nine participating countries could provide the required data, although not all in the required format. The data could be used to produce the surveillance tables as agreed in the reporting protocol. Most countries have the technical possibility of linking laboratory and epidemiological data; however, legal barriers for data sharing need to be clarified and agreement on a data collection protocol is required before routine HIV surveillance in the EU/EEA can be enhanced with HIVDR information.
